# Child Abuse in Group of Children with Attention Deficit-Hyperactivity Disorder in Comparison with Normal Children

**Published:** 2014-04

**Authors:** Habib Hadianfard

**Affiliations:** Department of Clinical Psychology, Shiraz University, Shiraz Iran

**Keywords:** Attention Deficit Hyperactivity Disorder, Child Abuse, Psychological Abuse, Neglect

## Abstract

**Background: **Children suffer from attention deficit hyperactivity disorder (ADHD) are very difficult to handle. It can be very frustrating and needs an outstanding tolerance. Behavioral difficulties in ADHD children may increase the risk of child abuse for them. The aim of this research was to compare child abuse, and neglect between ADHD group and normal children.

**Methods: **In this cross-sectional study, 30 ADHD students (10 girls and 20 boys) were selected from regional mental behavior disorder clinics and matched with 30 normal students. Data were collected using Child Abuse Self Report Scale. Descriptive statistics, one-way multivariate analysis of variance (MANOVA) and Least Significant Difference (LSD) was performed by using SPSS software.

**Result: **The result of the research showed that almost 60% of participants had experienced neglect and 35% psychological abuse. Neglect and psychological abuse are more frequent than other maltreatments. Neglect, psychological and physical abuses are significantly higher in the ADHD group.

**Conclusion: **The findings showed that the rates of neglect and psychological abuse are higher in the ADHD group. Therefore, it can be suggested that the society and families should be trained to deal better with ADHD children.

## Introduction

Attention deficit hyperactivity disorder (ADHD) is a neurodevelopmental disorder with symptoms which can be divided into two domains, inattention and impulsivity/hyperactivity.^1^ A child with ADHD persistently and repetitively shows social maladaptive behaviors such as temper tantrums, verbal and physical aggression, hostility, restlessness and externalizing behavior. Behavioral difficulties in ADHD such as hostile-intrusive behavior, bossiness, and impulsiveness almost interfere with the basic rights of others at school and home. As a result, these children have lots of conflict with teachers, classmates and parents.^2^ADHD children are frequently careless about rules and restrictions that set by adults; therefore, parents, teachers or other students may feel tired and helpless in dealing with them. For example, other children may not want to play with them at school or teachers ignore them easily in the classroom or among other students.^3^ Many teachers are not qualified for working with special needs children. They have not been trained to manage behavioral difficulties in children. A study in New Zealand showed that teachers accepted the fact that they have not enough knowledge about dealing with special needs children.^4^

Genetic research shows that ADHD is mainly a heritable disorder.^5^ It means many children with ADHD have parents suffering from adult ADHD. Children whose parents are suffering from mental health disorders receive poor parenting.^6^ Research shows that adults with ADHD have difficulty parenting. For example, they experience more stress,^7^ they get frustrated easier, argue with family members more, and have lower self confidence.^8^ On the other hand, general public, especially teachers and parents, commonly are not familiar with ADHD.^9^ They do not consider ADHD as a mental disorder.^10^ They always use the phrase “Boys are boys” as an excuse for accepting behaviours linked with ADHD. For example, 36% of a research participants had not heard about ADHD at all and 22% of those who had heard about ADHD believed ADHD is not a “real” disease.^10^ In general, when people have negative stereotypes, beliefs and attitudes about mental health or they are not able to recognize a mental problem correctly, they will respond inappropriately to the situation. Attitudes towards mental disorders are often important factors for seeking treatment. People who recognize mental health will manage or prevent it more quickly and effectively also they are more optimistic about the treatment outcomes. The term Mental Health Literacy has been defined as a person’s sufficient knowledge about a specific disorder and the skills he/she has about the interaction with the patient.^11^^,^^12^ Public knowledge about ADHD is important because it shows the public perception about ADHD and the way they interact with such cases. As a result, only half of children with ADHD receive medical treatment.^13^ This is even worse in developing countries.^14^ Because of disregarding the ADHD behaviours, some parents are unable to help or communicate correctly with an ADHD person. Some researchers have shown that fathers of children with ADHD show a strong resistance for treatment of ADHD.^15^^-^^17^ Sometimes parents have incorrect beliefs about the method of interventions. There are a number of facts that people who do not have mental health literacy; they probably behave more imperfectly toward others who have mental health problems.^18^ The combination of ADHD child’s temperament problems, the residual symptoms of ADHD in parents, and mental health illiteracy cause the family members to develop ineffective communication and aggressive relationship with others.^19^ Effective communication and healthy relationships within the family are very important because it enables the members to communicate with each other in a secure and respectful way. On the other hand, impulsiveness and externalizing problems cause unhealthy communication and child maltreatment in the family.^20^ Child abuse is a common problem in the world and in Iran.^21^ According to National Center for Juvenile in 1993, about 3 million children in the United States of America were abused. Nearly half of them experienced serious or moderate injury for that reason.^22^ The results of a study in Tehran showed that the prevalence of child abuse among students of district 20 of Tehran School Board was between 37% and 46%.  Reuters also reported a prevalence rate between 16% and 24%   for district 3 of Tehran School Board.^23^ Child maltreatment occurs in many different forms. National Society for Prevention of Cruelty to Children (NSPCC) defines the types of child abuse. a) Physical abuse: which involves every action that causes physical injury to a child b) Emotional Abuse: which involves every action that affects the child’s self-esteem negatively. c) Neglect: which involves ignoring the child’s essential needs (physical or psychological).^24^ Evidence shows that children with mental health and developmental problems are at risk of physical abuse and neglect.^25^ For example, 18.5% of children with autism had been physically abused^26^ or 14.3% of girls with ADHD are at risk of abuse. These rates are significantly higher in comparison with other groups.^27^ Parents are one of the major sources of child abuse in Iran.^23^

As the parent-child relationship is highly affected by cultural habits, cultural beliefs and cultural mental health literacy, the aim of this research was to examine whether Iranian children with attention-deficit/hyperactivity disorder (ADHD) are more at risk of child abuse and neglect in comparison with normal children. 

## Materials and Methods

The methodology applied in the present research is a cross-sectional one with two groups of ADHD and normal. The ethical aspect of the study was approved by the research council of the regional school board and oral consent was obtained from each subject. Subjects in both groups were matched for sex, age and grade of study. Data were collected over a period of four months. Each subject completed the Child Abuse Self Report Scale by the help of an assistant psychologist separately. After establishing the rapport, the assistant read clearly each item of the questionnaire for the participant and helped the subject to record the answers.

Thirty ADHD students (10 girls and 20 boys) aged 7-12 were selected through a systematic sampling from the list of clients who were registered for receiving cognitive behavioral intervention in one of the children’s behavior disorder clinics of Shiraz school board from September to December of 2011. The subjects were included in the ADHD group when his/her file showed he/she was diagnosed as ADHD by a psychiatrist, after meeting the DSM-IV criteria for ADHD. We excluded the participants with a current major depression, bipolar disorder, psychotic disorders, a lifetime history of traumatic brain injury, autistic disorder, and mental retardation. 

Thirty control subjects (10 girls and 20 boys) were recruited through multistage cluster sampling from Shiraz elementary public schools. To recruit the sample, we first randomly selected one girls’ and one boys’ public school from the list of public schools in each region of the Shiraz school board (Shiraz school board has four regions), and then 30 students were selected from the selected schools randomly.

The subjects were included in the normal group when they had no history of mental health problems. There was no significant difference between the ages of both groups (P>0.00). Table 1 shows some demographic characteristics of the participants. Although the researcher did not match the parents’ age, the mean ages of the parents were very close in both groups.

**Table 1:** Mean  and standard deviation of some demographic characteristics of participants

	**Age of participants**	**Age of fathers **	**Age of mothers **	**Number of siblings**
ADHD group	9.63 (1.15)	40.27 (6.47)	34.9 (5.83)	2.23 (1.56)
Normal Group	9.60 (1.16)	41.37 (5.75)	36.10 (5.89)	2.63 (1.09)
t value	0.11	0.696	0.793	1.14
P value	0.946 (NS)	0.529 (NS)	0.671 (NS)	0.405 (NS)

Data were collected using Child Abuse Self Report Scale (CASRS).^28^ The writers made CASRS for use in Iran. They showed that the test is a good tool for measuring child abuse in Iran. CASRS is a 38-item self-report questionnaire measuring four areas of abuse.  Each response is rated at four points of Likert scale ranging from never to always. The  authors who made the test reported its internal consistency between 0.87 and 0.95 and the test-retest reliability of the scales between 0.82 and 0.89. They also reported strong discrimination ability, and convergent and construct validity for CASRS.^28^ Each item is scored from 0-3. A score of “0” is given for no abuse or neglect responses and a score of “3” is given for severe abuse or neglect. As the number of items in each subtest is different, the users calculate the mean of each subtest; thus, the scores for the test and each subtest range from 0-3. In addition, the researcher collected some demographic data including age, gender, parents’ education and parent s’ age.

Descriptive statistics were used to describe the percentage of child abuse. The severity of abuse was classified into three levels based on each participant’s mean. When the average score of a subject was under 0.75, she/he was classified into the no abuse group. In case the average was over 1.75, we classified the subject into the moderate abuse group. Scores between 0.76 and 1.74 were put in the mild abuse group. We held each type of child abuse as a dependant variable and to test significant differences in child abuse between groups a one-way multivariate analysis of variance (MANOVA) was performed. To follow-up after rejecting null hypothesis or post-hoc test, we applied the Least Significant Difference (LSD) procedure for the matched groups. Data analysis was performed using SPSS 16 version. 

## Results

Table 2 shows the percentage of maltreatment based of the severity of abuse. 76.7% of the subjects in the ADHD group had experienced neglect. Neglect was more prevalent than the other types of abuse for all subjects and psychological abuse was the second frequent abuse.

**Table 2:** Percentage of child abuse

		**No abuse**	**Mild abuse**	**Moderate abuse**
Psychological abuse	All subjects	65%	26. 7%	8.3%
	ADHD group	46.7%	40%	13.3%
	Normal group	83.3%	13.3%	3.4%
Physical abuse	All subjects	65%	28.3%	6.7%
	ADHD group	53.3%	36.7%	10%
	Normal group	67.7%	20%	3.3%
Neglect	All subjects	40%	51.7%	8.3%
	ADHD group	23.3%	60%	16.7%
	Normal group	56.7%	43.3%	0
Total score	All subjects	61.7%	35%	3.3%
	ADHD group	43.3%	50%	6.7%
	Normal group	80%	20%	0

Multivariate analysis of variance (MONOVA) was used to analyze the data. As table 3 reveals, there is at least one significant difference between the two groups in one of the dependent variables.

**Table 3:** Multivariate tests

**Test**	**value**	**f**	**P value**
Pillai’s Trace	0.209	3.630	0.01
Wilk’s Lambda	0.791	3.630	0.01
Hotelling’s trace	0.264	3.630	0.01

As a follow up, ANOVA with pair-wise comparisons using the LSD procedure was applied to determine which of the possible comparisons between the means of the two groups was indeed statistically significant.  The result is shown in table 4.

**Table 4:** Pair-wise comparisons between ADHD and normal groups

	**Psychological abuse**	**Physical abuse**	**Neglect**
ADHD group	0.733	0.562	0.978
Normal Group	0.302	0.270	0.484
Mean Difference	0.431	0.292	0.494
P value	0.003	0.019	0.001

ADHD children in comparison with normal children had experienced more psychological, physical and neglect maltreatment. It means, ADHD persons were more likely to be a victim of abuse than normal children. 

## Discussion

The current result showed that many participants are subject to neglect and psychological abuse in their lives.  The frequency of such abuse for them is higher than other types of abuse. Nearly 60% of participant experienced neglect and 35% psychological abuse. These rates are higher than those already reported. Depending on the definition and measurement tools, the prevalence of psychological abuse was reported between 0.69 to 25.7 percent of children.^29^^,^^30^ A meta-analysis research with the use of 13 independent samples provided a global estimate of the prevalence of neglect. They reported 16.3% for physical neglect and 18.4% for emotional neglect.^30^ Psychological abuse and neglect are linked to each other and can be categorized as one item.^31^ Some researchers define two subtypes of neglect. Physical neglect refers to the ignorance of children’s physical needs, such as providing sufficient food, clothing, and care. Emotional neglect refers to ignorance of children’s emotional needs, such as providing adequate attention and affection.^32^ Emotional abuse and neglect are the most hidden and underestimated form of child maltreatment because there is no simple definition of them and they do not leave any physical traces on the victims. As a result, many offenders do not believe that they have abusive behaviour. They point out that their behavior was not intentional and they were weakly equipped with parenting information to manage efficiently children’s demands.^33^ Some researchers indicated that emotional abusive parents did not know how they can deal effectively with children they showed fewer coping skills, fewer child supervision strategies, and more trouble in establishing a relationship.^33^ It is unfortunate that less parenting literacy is more likely to be involved in child maltreatment. The results revealed a significant difference between the total score of abuse in the two groups of ADHD and normal. Further results of this study indicate that children with ADHD received significantly more maltreatment than the normal group. There are some reasons for this result. ADHD parents may be completely unaware of the effect of psychological abuse on children, so they use a strict discipline when they are under stress or tired of their children’s behavior. Another reason is lack of parenting skills particularly lack of knowledge about alternative positive behavior.

The result of the current research suggests that we have to work on primary prevention strategies in the field of child abuse before it occurs. Since emotional abuse may occur in very subtle varieties, primary prevention is very difficult. Two common interventions, such as parent education and home visiting programs, are suggested.^34^ The majority of parents and caregivers have limited knowledge about children with special needs. They have not learned coping strategies to help to communicate and deal with ADHD children. They need to achieve some parenting education, especially on non-violent interventions. A parent educational program is a systematic workshop that is run by a mental health professional including a psychologist, psychiatrist and psychiatric nurses aiming at improving one’s parenting skills (such as behavioral management techniques, problem solving, and personal coping skills) while he/she is working with ADHD children. The other aims of the parent educational programs are increasing self-confidence of the parents and reducing parental stress.^35^

The aim of a home visiting program is to improve the wellbeing of the child and family by assessing family problems and resources. Home visiting program is run almost by a nurse. A home visitor helps family members to move ahead to reach self efficacy, effective communication and decrease the risk of child maltreatment. Sometimes she may refer them for other supports or professional aids.^36^

## Conclusion

Based on the findings of this research, we can conclude that ADHD children are more likely to be victims of violence and neglect. They receive more psychological abuse and neglect. Therefore, we suggest mental health education and training for the society and families. 
